# Clinical results after external reinforcement of colorectal anastomosis: a systematic review

**DOI:** 10.1097/JS9.0000000000000747

**Published:** 2023-09-13

**Authors:** Clara Gené-Škrabec, Manel Cremades, Andrea Fernández-Pujol, Sara Cortinovis, Javier Corral, Joan-F Julián, David Parés

**Affiliations:** a Department of General Surgery, Hospital Universitari Germans Trias i Pujol, School of Medicine, Universitat Autònoma de Barcelona, Institut de Recerca Germans Trias i Pujol – IGTP, Barcelona, Spain; b Department of General Surgery, King’s College London, London, England; c Department of General Surgery, ASUGI Cattinara, Università degli studi di Trieste, Trieste, Italy

**Keywords:** anastomotic leak, external coating, sealant

## Abstract

**Objective:**

The aim of this review is to describe and assess the existing methods to cover colorectal anastomoses with biomaterials and their clinical impact in reducing anastomotic leakage (AL).

**Summary background data:**

The most serious complication in colorectal surgery is AL. Despite improvements in its diagnosis and management, AL remains an unresolved issue. To prevent its appearance and clinical consequences, different external reinforcement techniques with synthetic or biomaterials have been described.

**Methods:**

A systematic review search of the available literature until June 2022 was performed, looking for all literature regarding external reinforcement of colonic or colorectal anastomoses. After the review process, a classification of materials was proposed into solid and liquid materials, and an assessment of their clinical impact was performed. The study protocol has been registered at PROSPERO and has been reported in the line with PRISMA and AMSTAR Guidelines^11,12^.

**Results:**

Ninety-seven articles that fulfilled inclusion criteria, were identified and revised. Overall, 18 of the selected articles focused on human clinical trials and 79 on animal models. Only fibrin sealants, collagen patches, and omentoplasty have shown positive results in humans.

**Conclusions:**

Fibrin sealants, collagen patches, and omentoplasty are, so far, the most studied biomaterials. However, further studies are required to confirm these findings before definite recommendations can be made.

## Introduction

Highlights

- There are several methods to cover colorectal anastomoses with biomaterials to avoid postoperative anastomotic leak.
- Only omentoplasty, collagen patches, and fibrin sealants showed clinical advantages in humans to reduce anastomotic leak.
- However, definitive or strong recommendations cannot be made with the quality of data available.

Colorectal surgery often requires an anastomosis, both in cancer procedures and in benign conditions, like inflammatory bowel disease or diverticulitis. Thus, the most dreaded complication that colorectal surgeons face is the development of an anastomotic leak (AL). AL remains an unresolved problem and reducing its appearance or its clinical impact is essential.

The most broadly accepted definition of AL was proposed in 2010 by the International Study Group of Rectal Cancer (ISREC). It consists in a defect of the intestinal wall’s integrity at the anastomotic site leading to a communication between intraluminal and extraluminal compartments^1,2^. A pelvic abscess adjacent to the anastomosis is also considered an AL (with or without demonstrated communication). Although this definition was developed for low anterior resections, it is widely accepted for all colorectal anastomosis^3^.

The incidence of AL in colorectal surgery varies greatly depending on the location of the anastomosis, ranging from 1 to 20%^2^. Left-sided colonic and colorectal anastomoses have the highest failure rates. The consequences of an AL have been thoroughly described such as morbidity, higher mortality, poor quality of life, higher chances of a permanent stoma, local and distal recurrence of cancer, and a high economic burden^2^.

There are multiple factors described that contribute to AL. A tension-free, properly vascularized anastomosis is essential, but even in a technically perfect anastomosis, leaks might still appear. Efforts have been made to develop predictive scores, identify risk factors and to assess blood supply, fecal microbiome, and other agents that might be involved in AL. At present, the focus is set on detecting biochemical markers to identify AL as soon as possible as it can be handled less aggressively than when it is clinically evident^4,5^. Even though patient-related risk factors such as age, comorbidities, drug use, and nutritional status, cannot be modified, surgeons may have an opportunity to act considering technical factors. In fact, some authors have found that a surgeon’s expertise is one of the key factors in AL^6^.

One of the proposed methods to decrease AL is reinforcing anastomoses with an external coating. A review about this topic was published by Pommergaard *et al*.^7^ 10 years ago, where he stated that external coating had yet to demonstrate convincing results and urged for RCT and high-quality experimental studies to prove the clinical benefit of fibrin sealants (FS), omentoplasty (OP), and other coating materials.

At the moment, several other techniques of anastomotic reinforcement such as compression rings^8^, double layer-stitches, triple-line staplers or buttressing bioabsorbable materials in the stapler line or internal colonic sheets^9,10^ have been described. However, they are not reported in this review, as this review’s main aim and focus is to thoroughly assess the state of the art about external covering of colorectal anastomoses.

## Methods

A systematic review of RCT and nonrandomized human and animal trials was performed following a detailed protocol according to the PRISMA checklist^11^ (Supplemental Digital Content 1, http://links.lww.com/JS9/A995). The study protocol has been registered at PROSPERO and has been reported in the line with PRISMA and AMSTAR Guidelines (Supplemental Digital Content 2, http://links.lww.com/JS9/A996)^12^.

### Search strategy and information sources

A literature search published before June 2022 was conducted using the MEDLINE database via PubMed, Embase, and the Cochrane library and a manual search from Google Scholar. The used search terms were: anastomotic leak, coating, external, reinforcement, buttressing, wrapping, colorectal anastomosis (for the complete search strategy see PROSPERO protocol). Relevant articles from reviewed citations were also retrieved. Two reviewers (C.G., M.C.) evaluated the titles and abstracts of the articles focusing on colorectal anastomosis and its external reinforcement by a biomaterial, and a third reviewer (D.P.) solved any discrepancies about inclusion. Following this, a full-text paper evaluation was performed.

### Inclusion and exclusion criteria

#### Inclusion criteria

Articles reporting stapled or handsewn anastomosis involving the colon or rectum conducted either in live animals or humans, that had as their main focus or endpoint the avoidance of leakage by externally reinforcing the anastomosis.

#### Exclusion criteria

Articles reporting solely other locations of intestinal anastomoses, studies evaluating coating material placed internally or evaluating the coating of sutureless anastomosis. Articles in a language other than English or Spanish. Handsewn reinforcement procedures were not included as it was deemed as a modification of an anastomotic technique and not a proper external coating of the anastomosis.

### Outcomes

The primary endpoint of interest was to evaluate the incidence of AL in colorectal surgery when using an external coating. Additional secondary goals included the assessment of morbidity and mortality.

Articles were classified according to the type of reinforcement into solid or fluid materials to report and describe the identified articles. Colonic and rectal anastomosis were also assessed separately, as well as animal and human studies.

### Quality methodology assessment

The quality of the retrieved human trials was assessed with a validated methodology quality score (MINCIR score)^13^ The risk of bias of the included comparative clinical studies was assessed using the Cochrane Risk of Bias tools, ROB-2^14^ tool for RCT and ROBINS-I^15^ for nonrandomized trials. Graphic visualization of the risk of bias was achieved through robvis tool^16^ (see Supplemental Digital Content (SDC) 1, Supplemental Digital Content 3, http://links.lww.com/JS9/A997 and 2, Supplemental Digital Content 4, http://links.lww.com/JS9/A998). Two investigators (M.C. and C.G.) completed the quality and bias assessment independently and blinded to each other’s result. The discrepancy between evaluations was solved by a third reviewer (D.P.).

## Results

**Figure 1:**
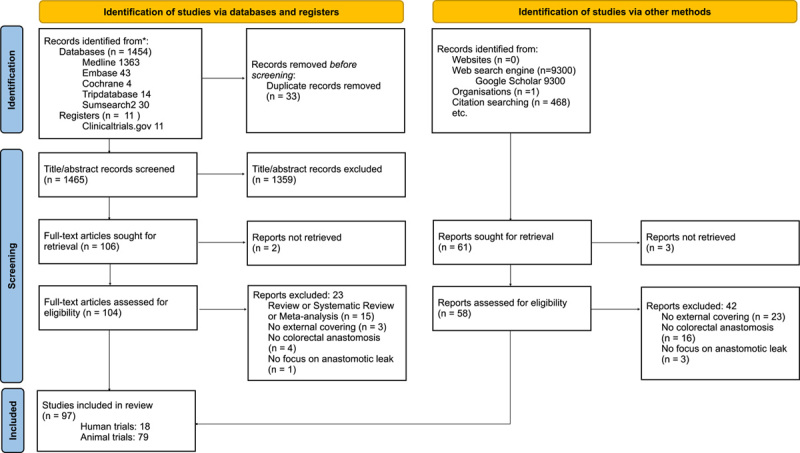
Flowchart of the selected articles.

**Figure 2:**
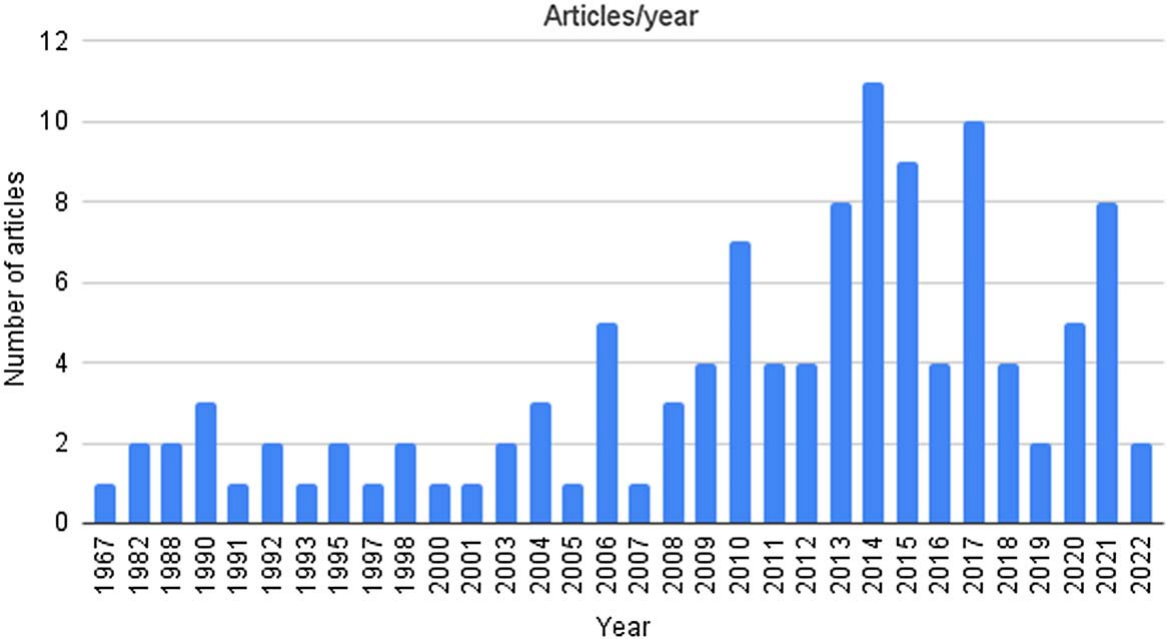
Bibliometric results.

Ninety-seven articles were identified and revised, 18 articles were human clinical trials (7 randomized clinical trials and 11 case reports), and 79 articles were animal trials (see Fig. 1 Flowchart). Fifteen revisions about this topic were also identified. Although they were reviewed and used for reference citation, they were not included in the final analysis. The majority of articles about this topic have been published in the last 15 years (see Fig. 2 Bibliometric results). The data of the included studies were very heterogeneous, preventing a meta-analysis, so the author’s opted for a narrative approach for this review.

The authors have classified the agents used to externally cover the colorectal anastomosis into 11 groups regarding their composition and differentiating between fluid and solid categories. These agents, their commercial names, and their approved use cases are described in Table 1.

**Table 1:** Materials identified and their commercial names.

Liquid					
Commercial name	Manufactured by	Composition	Fibrinogen concentration	Approved uses	References
Fibrin sealant					
Tisseel (Tissucol)	Baxter Healthcare, Westlake Village, CA, USA	Human derived Fibrinogen and Thrombin	67–110 mg/ml	Hemostatic and tissue sealing	^17–19^
Greenplast	Green Cross, Seoul, South Korea	Fibrinogen, factor XIII and thrombin	90 mg/ml	Hemostatic	^18^
Evicel	Omrix Biopharmaceuticals N. V., Diegem, Belgium	Human fibrinogen and fibronectin and thrombin	55–85 mg/ml	Hemostatic	^20,21^
PRP (Platelet rich plasma)					
Obsidian ASG (Anastomotic SafeGuard)	Revolution GmbH, Rosenheim, Germany and Vivostat A/S, Alleroed, Denmark	Autologous bioactive fibrin sealant combined with an autologous platelet concentrate		Colorectal suture reinforcement	^22^
Cyanoacrylates					
Histoacryl flex	B Braun. Melsunger, Germany	N-butyl-cyanoacrylate (enbucrilate)		Tissular adhesive, sclerotherapy and mesh fixation	^23,24^
Histoacryl	B Braun. Melsunger, Germany	N-butyl-cyanoacrylate (enbucrilate)		Skin closure, sclerotherapy and mesh fixation	^23^
Omnex	Ethicon, Somerville, NJ, USA	2-octyl-cyanoacrylate and butyl-lactoyl-cyanoacrylate		Hemostatic	^20,25^
Glubrand 2	GEM slr, Viareggio LU, Italy	N-butyl-cyanoacrylate and monomer		Hemostatic, vascular embolization, mesh fixation and tissue adhesive	^26^
Exofin	Chemence Medical Inc., Alpharreta GA, USA	2-octyl- cyanoacrrylate		Skin closure	^27^
GluSeal	Skinstitch Corp. Delta, BC, Canada	2-octyl- cyanoacrrylate		Skin closure	^28^
Dermabond	Ethicon, Somerville, NJ, USA	2-octyl- cyanoacrrylate		Skin closure	^29^
Polyethylene glycol (PEG)					
Coseal	Baxter Healthcare, Deerfield, IL, USA	Polyethylene glycol and liquid sodium phosphate buffer		Reinforcement of vascular sutures and lung resections. Prevention of adhesions	^30^
Duraseal	Covidien. Waltham, MA, USA	Polyethylene glycol and trysilyn- amine		Dural sealant in neurosurgery	^23,24,31^
VascuSeal	Covidien. Waltham, MA, USA	Synthetic polyethylene glycol-based hydrogel sealant.		Tissue seal and space filling	^20^
PleuraSeal	Covidien, Mansfield, MA, USA	Synthetic polyethylene glycol-based hydrogel sealant		Prevention of air leak after lung resection	^20^
Albumin based sealants					
BioGlue	CryoLife, Inc. Kennesaw, GA, USA	Purified bovine serum albumin (BSA) and glutaraldehyde		Hemostasis (vascular surgery)	^31^
Colle Chirurgicale Cardia	LeMaitre Cardial, Saint-Etienne, France	Gelatin, resorcinol, formaldehyde and glutaraldehyde		Reinforce sutures in vascular anastomoses	^19^
Gelatine solution					
GRF Glue	Cardial SA. St Ettiene, France	Gelatin, Resorcin, and Formalin		Aortic and cardiac surgery	^32^
Lifeseal	LifeBond Ltd. Caesarea, Israel	Gelatin solution and PEGylated purified mTG solution		Stapler-line reinforcement	^20^
Polysaccharides and phospholipids					
Marine inspired hydrogel	TCI Development Co., Ltd, Japan./ Aladdin Co., Ltd, China	Dopamine-conjugated xanthan gum (Da-g-Xan)		Skin closure and gastrointestinal anastomoses	^34^
Pebisut	Maxweel SA and Pebisut, Mexico	Carbohydrate and zinc		Coadjuvant for healing of anastomoses	^35^
PVP-I liposome hydrogel	Mundipharma GmBH, Limburg, Germany	Liposomal polyvinylpyrrolidone (PVP)-iodine		Common inflammatory skin disorders	^36^
SOLID					
Commercial name	Manufactured by	Composition	Fibrinogen concentration	Approved uses	References
Collagen patches					
Tachosil	Corza Mecial GmBH, Dusseldorf, Germany	Human fibrinogen and thrombin	5.5 mg/cm^2^	Hemostatic, support stitching, prevent leakage in neurosurgery	^37–43^
Hemopatch	Baxter Healthcare, Deerfield, IL, USA	Collagen matrix and NHS-PEG (polyethylene glycol)		Hemostatic and surgical sealant (liquid, air and dural)	^44^
Bio-Gide	Geistlich. Wolhusen, Switzerland	Porcine collagen type I and II		Oral tissue regeneration	^45^
Surgisis/Biodesign	Cook Medical, Bloomington, Indiana, US	Porcine small intestine submucosa		Hiatal hernia grafts, stapler line reinforcement, anal fistula plug	^45,46^
Tutomesh	Tutogen Medical GmbH, Neunkirchen am Brand, Germany	Non-crosslinked acellular collagen I matrices derived from bovine pericardium		Breast surgery, hernia repair and gastrointestinal anastomosis	^47–49^
Polysaccharides					
Seprafilm	Baxter Healthcare, Deerfield, IL, USA	Modified sodium hyaluronate (HA) and carboxymethylcellulose (CMC)		Abdominal surgery adhesion prevention	^50^
Polymers					
Tissuepatch	Tissumed Ltd, Leeds, UK	Alternate layers of poly(lactide-co-glycolide) and poly (N-vinyl-pyrrolidone50-co-acrylic acid 25- co-N-hydroxysuccinimide ester of acrylic acid 25)		Seal or reinforce against air leakage	^51^
Vicryl	Ethicon, Somerville, NJ, USA	Polyglactin 910 (PGA) mesh		Hernia surgery	^52^
Prolene	Ethicon, Somerville, NJ, USA	Polypropylene mesh		Hernia surgery	^53^
Nanofibers		Blow spun or electro-spun polymers. Typically, Polycaprolactone (PCL) and a polylactic acid-polycaprolactone copolymer		Fortification of intestinal anastomoses	^54–56^
Others biological materials used	References				
Omentum	^40,57–62^				
Mesenteric flap	^63^				
Peritoneal graft	^37^				
Amniotic membrane	^64^				
Stem cells	^65,66^				

To focus on our primary endpoint, human clinical trials are described in detail in Table 2. Only six materials have been tested in humans and of these. OP and FSs have shown significant positive results in reducing AL rates (see Table 2). All RCT had a control group with no intervention, as well as the rest of the nonrandomized studies, except for AAlbantonya^70^ and Hegab^40^ that compared OP and FG coated collagen patch. Five articles were a series of cases with no control group^22,39,44,51,63^.

**Table 2:** Summary of human clinical trials found through this review and their characteristics.

Coating material (^a^)	Year	Author	Design	MIN-CIR	Anastomotic site	Surgical technique	Surgery indication	Anastomotic technique	N exp. (Total)	AL study %	AL control %	Mortality study %	Mortality control %	Morbidity study %	Morbidity control %
Fibrin sealant	2010^18^	Huh	CR	15	Rectum	Laparoscopic	Cancer	Mechanical	104 (223)	5.8	10.9	NR	NR	15.4	16.8
Fibrin sealant (Tisseel)	2012^17^	Lago Oliver	RCT	35	Rectum/Colon/Upper GI	Open/Laparoscopic	Other	NR	17 (104)	**13.4**	**28.8** ^*^	5.7	7.7	NR	NR
Fibrin sealant (Tissucol or Greenplast)	2014^67^	Kim	CR	16	Rectum	Open/Laparoscopic	Cancer	Manual/Mechanical	328 (1148)	4.3	5.8	NR	NR	NR	NR
Fibrin sealant	2015^68^	Sieda	CR	17	Colon	Open	Cancer, other	Manual	35 (70)	20	8.6	NR	NR	5.7	11.4
Fibrin sealant (Tisseel)	2015^69^	Lago Oliver	RCT	22	Rectum/Sigmoid colon	Open/Laparoscopic	Cancer	Mechanical	16 (37)	**18.7**	**52.4** ^*^	6.3	19	**12.5**	**42.9** ^*^
PRP (Obsidian ASG)	2021^22^	Shamiyeh	CR	15	Left Colon/Rectum	Laparoscopic/Robotic	Cancer	Mechanical	261 (261)	2.3	—	0	—	4.5–15.5%	—
Collagen patch (Tachosil)	2013^39^	Parker	CR	14	Colon/Rectum	Open/Laparoscopic	Cancer, Diverticular, other	Mechanical	25 (25)	8	—	NR	—	NR	—
Collagen patch (Tachosil)	2016^43^	Torres-Melero	CR	16	Colon	Open	Debulking,peritoneal carcinomatosis	Mechanical	22 (49)	0	6.1	NR	NR	NR	NR
Collagen patch (Hemopatch)	2021^44^	Kornfeld	CR	6	Rectum	Open	Cancer	Mechanical	8 (10)	12.5	—	NR	—	NR	—
Polysaccharides (Seprafilm)	2003^50^	Beck	RCT	35	Colon/Rectum	Open	Diverticular, IBD, other	Manual	289 (1791)	**6.9**	**2.4** ^*^	NR	NR	NR	NR
Polymer (Tissue patch)^**^	2018^51^	Trotter	CR	14	Colon	Open/Laparoscopic	Cancer, Diverticular, other	Manual/Mechanical	9 (9)	86	—	0	—	88	—
Omentoplasty	1998^58^	Merad	RCT	36	Colon/Rectum	NR	Cancer, Diverticular, IBD, other	Manual	341 (712)	4.7	5.2	4.9	4.2	9	8
Omentoplasty	2000^59^	Tocchi	RCT	27	Rectum	Open	Cancer	Mechanical	53 (115)	**3.8**	**11.8** ^*^	NR	NR	NR	NR
Omentoplasty	2004^60^	Agnifilli	RCT	19	Rectum	NR	Cancer, Diverticular, other	NR	62 (126)	**6.4**	**21.9** ^*^	**3.2**	**7.8** ^*^	NR	NR
Omentoplasty – Collagen patch	2014^70^	AAlbatanonya	CR	13	Colon/Rectum	NR	NR	Manual/Mechanical	48 (97)	8.3	2	2	0	27	28
Omentoplasty – Collagen patch	2016^40^	Hegab	CR	12	Colon/Rectum	NR	Cancer	Manual/Mechanical	48 (97)	8.3	2	2	0	27	28
Omentoplasty	2017^61^	Nasiri	RCT	21	Small bowel/Colon/Rectal	NR	Cancer, other	Manual/Mechanical	62 (124)	**4.8**	**14,5** ^*^	0	9.7	**4.8**	**14.5** ^*^
Mesenteric flap	2013^63^	Mohan	CR	6	Colon/Rectum	Open/Laparoscopic	NR	Manual/Mechanical	20 (20)	0	—	NR	—	NR	—

Table 2 Human tested materials and its characteristics

a When mentioned, the commercial name of the material is added in brackets.

* Statistically significant results are marked with a (*). When there is no comparison group, the values reported in the articles are mentioned.

** This trial was prematurely terminated because of suspected side effects.

CR, clinical trials or reports; IBD, inflammatory bowel disease; MIN-CIR, Quality assessment scale, see Methods; N exp. (Total), number of participants on the experimental arm (Total number of participants); NR, not reported; PRP, platelet rich plasma; RCT, randomized controlled trial.

Additional secondary goals (morbidity and mortality) were assessed but unfortunately, most of the articles did not report them.

Most of the reinforcement materials have been evaluated through animal trials. Five animal models have been used; the most used animal model is the rat followed by the pig, dog, rabbit, and mice models. To assess the list of animal studies found though this review (see SDC 3, Supplemental Digital Content 5, http://links.lww.com/JS9/A999. Materials tested in animal studies, Supplemental Digital Content 3, http://links.lww.com/JS9/A997).

A classification and detailed explanation of the most used groups of coating materials is detailed as follows:

### Reinforcement of colorectal anastomosis using fluid materials

#### Clinically tested materials

*Fibrin Sealant*: Undeniably, the most used and tested agent. Fibrin glue (FG) or FS comprises a big family of products, but they are all based on the principles of clot formation. FG is composed of two elements, fibrinogen and thrombin. Triggered by bleeding, a leaking surface or physiological saline, thanks to the action of thrombin, fibrinogen is turned into fibrin monomers creating the fibrin clot. Thrombin can also activate endogenous factor XIII which cross-links the fibrin to create a firm and stable fibrin scaffold. This reaction requires the presence of small amounts of calcium and factor XIII^70^.

FG has been used as an active hemostatic agent for many years. It was approved by the Food and Drug Administration in 1998 and, since then, its indications have expanded, being currently also approved as a sealant and adhesive agent^10^. FG intrinsically carries a biologic risk due to its manufacturing from human plasma, making it possible to transmit infectious agents.

Many formulations exist. Some FSs have also been bound to patches or matrices (see Tachosil) or in combination with other materials^7,19^. Concentrations of fibrinogen and thrombin vary among the commercialized materials, following a table with the existing sealants tested in colorectal anastomosis (see Table 1).

#### Animal studies

FS has been tested in mice, rat, rabbit, dogs, and pig models with conflicting results in anastomotic healing. In 2014, Nordentoft *et al*.^71^ in a systematic review on animal studies and FS concluded that FS might have an influence on sealing anastomosis due to mechanical strength, but it does not have a consistent positive result on the healing of gastrointestinal anastomoses.

#### Human studies

FS has shown positive and significant results in reducing AL, in a couple of human RCTs. see Table 2 for detailed results.

So far, the only approved sealant with the indication of covering colonic anastomosis or the revision of stomas is Tisseel.

*Platelet Rich Plasma (PRP)*: PRP is a concentrated solution derived from patients’ serum obtained by centrifugation of the patient’s blood. It contains a higher-than-normal concentration of platelets as well as cytokines and growth factors naturally present in the plasma. PRP has been extensively used in orthopedic surgery and reconstructive surgery^10^.

#### Animal studies

In 2021, Geropoulos^72^ described in his systematic review and meta-analysis about 18 animal studies using PRP on bowel anastomosis that its application shows improved outcomes in terms of anastomoses bursting pressure and tissue hydroxyproline.

#### Human studies

Also in 2021, Shamiyeh^22^ tested PRP in humans in a clinical trial for the first time on colonic and rectal anastomosis, concluding that it is safe to use with a low rate of AL.

#### Experimentally tested materials

*Peg-Based Sealants*: Polyethylene glycol (PEG)-based sealants are novel synthetic tissue adhesives that combine different forms of PEG to form a hydrogel. One of their advantages of PEG-sealants is that they do not entail the biologic risks associated with fibrin glues or PRP. They are absorbable and noncytotoxic^25^. However, PEG-sealants can swell causing injury to neighboring tissues.

#### Animal studies

Slieker *et al*.^20^ tested two-types of PEGs, finding no evidence that these sealants reduce leakage rates in a mouse model. However, they state that a possible reduction of clinical AL could be possible and that further studies with large animal models are necessary.

#### Human studies

PEG-based sealants have never been tested on colorectal anastomoses in human studies.

*Cyanoacrylates*: Cyanoacrylates (CA) are a family of fast-acting adhesives that rapidly polymerize in the presence of moisture. They were initially invented for industrial purposes, but in 1998 the FDA approved its use in the medical field, when Dermabond was approved for topical-skin closure.

Chemically, the structure of the CA is made of three compounds. The ethylene group and cyano-group remain the same throughout the cyanoacrylate family but the third compound, the alkyl-group is what changes in each cyanoacrylate and gives the different properties to the glue. They can be butyl (N- or iso-), ethyl, methyl, or octyl CA (see Table 1).

Nowadays they are extensively used in skin wound closure, but their use on intestinal anastomosis is still under debate. In general, CA produce an exothermic reaction when they polymerize. This reaction is tamed by softeners and other chemical compounds. There are some concerns about toxicity. It is believed that CA toxicity is due to low reabsorption leading to a foreign-body reaction, but also due to the production and accumulation of formaldehyde and cyanoacetate (degradation products of CA). In their systematic review Wu *et al*.^24^ stated that CA application on intestinal and colorectal anastomosis seems promising.

#### Animal studies

CA have been tested in animal trials, in mice, rat and pig models with contradictory results^73^.

#### Human studies

CA have never been tested on colorectal anastomoses in human studies. For the moment, the results of the ongoing ReAL trial^74^ are yet to be known.

### Reinforcement of colorectal anastomosis using solid materials

#### Clinically tested materials

*Omentoplasty*: OP is a procedure that involves wrapping or approximating a pediculated portion of well-vascularized omentum to cover an area^9^. It has been used to cover gastric defects after ulcers or to fill out the pelvis after abdomino-perineal resection^75^.

#### Animal studies

Gulati *et al*.^76^ tested omental reinforcement in a canine model, concluding that both the omentum and peritoneum were not effective in reducing AL.

#### Human studies

This is the most extensively studied external reinforcement of the colorectal anastomosis technique in humans^70^. Although three meta-analyses have been written about the topic^57,62,77^ only four RCT have been carried out^58–61^. Hao *et al*.^77^ found no supportive evidence for OP. Wiggins *et al*.^62^ analyzed all GI anastomosis and commented on three color-rectal studies that found no differences in colorectal anastomosis when using OP in AL nor in hospital stay rates. Recently, Sahebally *et al*.^57^ stated that, although OP appears to reduce the rate of overall and clinical AL, the heterogeneity in the data does not allow definitive recommendations. In conclusion, the data about the use of OP for protecting colorectal anastomoses is conflicting. It can be safely used, but no clear beneficial effect can be confirmed so far.

*Collagen Patches*: Collagen patches are a group of agents used in numerous surgical specialties. They are often covered in other active substances and have been extensively used as hemostatic and for organ repair as they work as an optimal structural skeleton for the growth of fibroblasts. Pure collagen generates very few immunological reactions. After its placement, it is slowly enzymatically degraded and replaced by the body’s own connective tissue. As the body’s predominant type of collagen is type I, most of the patches are made of it. This group also comprises bovine pericardium, which is a popular material for dura replacement and so far, has only been used in experimental trials^47–49^. The two most used collagen patches for colonic anastomoses are described as follows:

#### Tachosil

Tachosil is the most used brand of collagen patch coated with active substances. It consists of an equine-derived collagen matrix binded to human fibrinogen and thrombin. It is intended to offer the benefits of a FS harnessing its properties into a patch. The approved indications are to promote hemostasis and in neurosurgery.

#### Animal studies

In animal studies, most of the reports are favorable^41,42^ Pommergaard *et al*.^44^ tested Tachosil in technically insufficient colon anastomoses on a mice model. They found a significant reduction of the number of leakages by Tachosil coating. However, more cases of large bowel obstruction were found in the Tachosil group.

#### Human studies

No significant positive results regarding Tachosil have been described. AAlbatanonya^70^ compared OP to Tachosil usage and concluded that the increase of average cost per patient by using Tachosil could be compensated by a shorter hospital stay as the Tachosil group had more favorable results. Tachosil was tested in 2013 by Parker *et al*.^39^ with positive but not significant results as it was deemed feasible to apply around colorectal anastomosis. In 2016 Torres-Melero^43^ used it in debulking surgeries to coat anastomosis also with positive results and in 2016 Hegab *et al*.^40^ found Tachosil more effective in preventing AL than OP. See Table 2 for detailed results.

#### Hemopatch

Hemopatch is a patch of collagen derived from bovine dermis, coated with NHS-PEG (pentaerythritol PEG ether tetra-succinimidyl glutarate). Its primary indication is as a hemostatic agent.

#### Animal studies

No animal studies using Hemopatch have been identified.

#### Human studies

In 2021, Kornfeld *et al*.^44^ described wrapping hemopatch around colorectal anastomosis with positive results, but they reported that the technique still requires standardization. At the moment, we are still awaiting the results of the ongoing RORA trial^78^.

*Seprafilm*: Seprafilm Adhesion Barrier is a bioresorbable, adherent, and translucent patch composed of two polysaccharides, sodium hyaluronate (HA) and carboxymethylcellulose (CMC)^50^.

#### Animal studies

In 1995, Medina *et al*.^79^ tested Seprafilm for the first time in a rabbit model concluding that HA/CMC is a potentially safe adjuvant for preventing postoperative intra-abdominal adhesions.

#### Human studies

In 2003, Beck *et al*.^50^ performed an RCT testing Seprafilm and in a subset of patients (*n*=289) it was also tested to reinforce colonic anastomosis. The study showed that Seprafilm was safe to use anywhere in the abdomen, but on an anastomosis. At the moment, the manufacturer states that Seprafilm should not be wrapped around a fresh suture or staple line as it increases the risk of AL. See Table 2 for detailed results.

#### Experimentally tested materials

*Nanofibers*: Nanofibrous materials are a wide array of fabrics created by polymeric biomaterials. Some types of nanofibrous biodegradable materials show a positive effect on wound healing, assumingly due to its structural similarities to collagenous extracellular matrix. They are often produced through electrospinning techniques allowing the creation of a nonwoven fabric. Among the most commonly used biomaterials for fabrication of nanofibrous scaffolds are: polycaprolactone, polylactide, polyglycolide, polydioxanone, polyhydroxybutyrate, and polyvinyl alcohol.

#### Animal studies

Between 2020 and 2021, Rosendorf *et al*. performed three experimental trials based on a pig model and demonstrated a positive result in the reduction of AL^54–56^.

#### Human studies

Nanofibers and blown spun polymers are yet to be tested in humans.

## Discussion

Anastomosis leak is one of the most troublesome complications in colorectal surgery with a clear clinical impact. Multiple factors influence the adequate healing of an anastomosis such as tension-free sutures, adequate vascularization, patient comorbidities and even so surgeon’s expertise. External coating of the anastomosis had been proposed as an additional technical part to further decrease AL rates, but it cannot be indeed, a solution for a nontechnically adequate performed anastomosis.

The vast majority of the studies reviewed regarding the external coating of anastomosis are experimental and very heterogeneous, very few studies are well-designed human RCT. Moreover, most of the tested materials have a different primary indication, as hemostatic or prevention of pelvic sepsis or adhesions instead of sealing anastomoses. Given the heterogeneity of the included studies, with diverse primary endpoints and the wide range of time of the publications and the subsequent risk of bias that the improvement on AL is due to technical improvement and not solely to the tested material, we have opted for a narrative approach for this review.

In this review, we intended to comprehensively classify the colorectal anastomosis. Different areas of the intestine and types of anastomoses have diverse leakage rates. A recent meta-analysis by Cira *et al*.^19^, focuses on all sutures of the GI tract, which does not allow us to confidently extrapolate the results into the colorectal setting. In our review, similar to Pommegaard’s systematic and comprehensive review^7^, we describe the external coating of colorectal anastomosis.

The definition of AL differs widely among studies, making comparisons again troublesome and misleading. Therefore, a clear definition of AL should be mentioned and a differentiation between clinical or radiological leak should also be made. Only a minority of authors stated these parameters. Abdominal bursting pressure, often assessed in animal trials, is probably not a relevant endpoint for clinical practice. Furthermore, when using a tissue adhesive, it is necessary to provide details on its application, such as the amount used, layer thickness, width, and time to let it set. In addition, most of the evaluated articles did not clearly report the potential and intuitive risk an external coating material might have such as: abscess formation, stenosis, or adhesions.

A coating material intended to be used in colorectal surgery, not only has to prove its benefits in preventing AL, but also the material should be easy to use. In a minimally invasive era (laparoscopic/robotic surgeries), a coating material may face a further drawback, as handling and applying a mesh or patch can be a technical issue. However, there is scarce information on this matter in published studies.

To the best of the author’s knowledge, this is the most thorough and complete review of external coating options on colorectal anastomosis. However, this study also has some limitations. The broadness of the topic and the heterogeneity of the retrieved articles did not allow us to perform a formal meta-analysis.

In summary, colon and rectal AL is a complex and multifactorial condition; research in any topic aiming to reduce its presentation is essential and relevant. Up until now, only OP, collagen patches and FS showed positive results in humans to reduce AL. However, further studies are required to confirm these findings before definite recommendations can be made.

## Ethical approval

Not applicable.

## Consent

Not applicable.

## Sources of funding

Part of the study has been funded with Grant TALENT -HUGTiP and FIS-ISCIII.

## Author contribution

C.G.Š.: design study, data analysis, co-write; M.C.P.: design study, data analysis, co-write; A.F.P., S.C., and J.C.R.: data analysis; J.-F.J.I.: design study, data analysis, co-write; D.P.: design study, data analysis, co-write, supervision.

## Conflicts of interest disclosure

No conflicts of interest.

## Research registration unique identifying number (UIN)

1. Name of the registry: PROSPERO registry.
2. Unique identifying number or registration ID: CRD42022312025.
3. Hyperlink to your specific registration (must be publicly accessible and will be checked): https://www.crd.york.ac.uk/prospero/display_record.php?RecordID=312025.

## Guarantor

Clara Gené Škrabec^1^, MD.

## Data availability statement

Not applicable.

## Provenance and peer review

Not commissioned, externally peer-reviewed.

## Supplementary Material

SUPPLEMENTARY MATERIAL
