# Supplementary material for: Clinical results after external reinforcement of colorectal anastomosis: a systematic review
Source: Int J Surg. 2023 Sep 13;109(12):4322–32. doi: 10.1097/JS9.0000000000000747 (PMC10720808; doi:10.1097/JS9.0000000000000747)
Supplement: SUPPLEMENTARY MATERIAL [file js9-109-4322-s003.docx]

SUPLEMMENTARY MATERIAL

SDC1. Materials tested in animal studies

| Animal model | Material | List of articles |
| --- | --- | --- |
| Mice | Fibrin sealant | ^1^ |
|  | Cyanoacrylates | ^1^ |
|  | Collagen patch | ^2–4^ |
|  | Nanofibers | ^5^ |
|  | Polysaccharides and phospholipids | ^6^ |
| Rat | Fibrin sealant | ^7–24^ |
|  | PRP | ^25–27^ |
|  | Cyanoacrylates | ^23,28–37^ |
|  | Albumin glue | ^38^ |
|  | Polysaccharides and phospholipids | ^6,39–41^ |
|  | Collagen patch | ^42–48^ |
|  | Polymers | ^49^ |
|  | Amniotic membrane | ^50^ |
|  | Stem cells | ^51,52^ |
| Rabbit | Fibrin sealant | ^53^ |
|  | PEG | ^54^ |
|  | Gelatin solution | ^55^ |
|  | Polysaccharides and phospholipids | ^56^ |
|  | Polymers | ^57^ |
| Dog | Fibrin sealant | ^58,59^ |
|  | Gelatin solution | ^55^ |
|  | Polysaccharides and phospholipids | ^40,60^ |
|  | Collagen patch | ^61,62^ |
|  | Omentoplasty | ^63^ |
|  | Other (polyurethane foam) | ^64^ |
| Pig | Fibrin sealant | ^65–67^ |
|  | PRP | ^68^ |
|  | Cyanoacrylates | ^69–71^ |
|  | Gelatin solution | ^72^ |
|  | Collagen patch | ^73–75^ |
|  | Nanofibers | ^5,76–78^ |
|  | Adipose stem cells | ^79^ |

LIST OF ARTICLES BASED ON ANIMAL TRIALS

1. Slieker JC, Vakalopoulos KA, Komen NA, et al. Prevention of leakage by sealing colon anastomosis: Experimental study in a mouse model. *Journal of Surgical Research*.;184 . Epub ahead of print 2013. DOI: 10.1016/j.jss.2013.04.015.

2. Pantelis D, Beissel A, Kahl P, et al. The effect of sealing with a fixed combination of collagen matrix-bound coagulation factors on the healing of colonic anastomoses in experimental high-risk mice models. *Langenbecks Arch Surg*.;395 . Epub ahead of print 2010. DOI: 10.1007/s00423-010-0703-5.

3. Nordentoft T, Holte K. Preventing Clinical Leakage of Colonic Anastomoses with A Fibrin-Coated Collagen Patch Sealing - An Experimental Study. *Archives of Clinical and Experimental Surgery (ACES)*.;3 . Epub ahead of print 2014. DOI: 10.5455/aces.20130321071651.

4. Pommergaard HC, Achiam MP, Burcharth J, et al. Decreased leakage rate of colonic anastomoses by tachosil coating: An experimental study. *Int Surg*.;99 . Epub ahead of print 2014. DOI: 10.9738/INTSURG-D-13-00093.1.

5. Kern NG, Behrens AM, Srinivasan P, et al. Solution blow spun polymer: A novel preclinical surgical sealant for bowel anastomoses. *J Pediatr Surg*.;52 . Epub ahead of print 2017. DOI: 10.1016/j.jpedsurg.2016.11.044.

6. Huang J, Jiang Y, Liu Y, et al. Marine-inspired molecular mimicry generates a drug-free, but immunogenic hydrogel adhesive protecting surgical anastomosis. *Bioact Mater*.;6 . Epub ahead of print 2021. DOI: 10.1016/j.bioactmat.2020.09.010.

7. Houston KA, Rotstein OD. Fibrin Sealant in High-Risk Colonic Anastomoses. *Archives of Surgery*.;123 . Epub ahead of print 1988. DOI: 10.1001/archsurg.1988.01400260118015.

8. Hulkko OA, Haukipuro KA, Laitinen ST. Fibrin glue protection of primary anastomosis in the obstructed left colon. An experimental study on the rat. *Acta Chir Scand*.;154.

9. van der Ham AC, Kort WJ, Weijma IM, et al. Effect of fibrin sealant on the healing colonic anastomosis in the rat. *British Journal of Surgery*.;78 . Epub ahead of print 1991. DOI: 10.1002/bjs.1800780117.

10. van der Ham AC, Kort WJ, Weijma IM, et al. Healing of ischemic colonic anastomosis: Fibrin sealant does not improve wound healing. *Dis Colon Rectum*.;35 . Epub ahead of print 1992. DOI: 10.1007/BF02047878.

11. Byrne DJ, Hardy J, Wood RAB, et al. Adverse influence of fibrin sealant on the healing of high-risk sutured colonic anastomoses. *J R Coll Surg Edinb*.;37.

12. van der Ham AC, Kort WJ, Weijma IM, et al. Transient protection of incomplete colonic anastomoses with fibrin sealant: An experimental study in the rat. *Journal of Surgical Research*.;55 . Epub ahead of print 1993. DOI: 10.1006/jsre.1993.1137.

13. Karahasanoglu T, Alcicek S, Altunkaya E, et al. Effect of fibrin glue on irradiated colonic anastomoses. *Dis Colon Rectum*.;40 . Epub ahead of print 1997. DOI: 10.1007/BF02055171.

14. Shinohara K, Kobayashi E, Yoshida T, et al. Effect of fibrin glue on small and large bowel anastomoses in the rat. *European Surgical Research*.;30 . Epub ahead of print 1998. DOI: 10.1159/000008552.

15. Kanellos I, Mantzoros I, Goulimaris I, et al. Effects of the use of fibrin glue around the colonic anastomosis of the rat. *Tech Coloproctol*.;7 . Epub ahead of print 2003. DOI: 10.1007/s10151-003-0014-8.

16. Kanellos I, Mantzoros I, Demetriades H, et al. Healing of Colon Anastomoses Covered with Fibrin Glue after Immediate Postoperative Intraperitoneal Administration of 5-Fluorouracil. *Dis Colon Rectum*.;47 . Epub ahead of print 2004. DOI: 10.1007/s10350-003-0085-7.

17. Li Y, Bao Y, Jiang T, et al. Effect of the combination of fibrin glue and growth hormone on incomplete intestinal anastomoses in a rat model of intra-abdominal sepsis. *Journal of Surgical Research*.;131 . Epub ahead of print 2006. DOI: 10.1016/j.jss.2005.09.013.

18. Akgün A, Kuru S, Uraldi C, et al. Early effects of fibrin sealant on colonic anastomosis in rats: An experimental and case-control study. *Tech Coloproctol*.;10 . Epub ahead of print 2006. DOI: 10.1007/s10151-006-0281-2.

19. Kanellos I, Christoforidis E, Kanellos D, et al. The healing of colon anastomosis covered with fibrin glue after early postoperative intraperitoneal chemotherapy. *Tech Coloproctol*.;10 . Epub ahead of print 2006. DOI: 10.1007/s10151-006-0263-4.

20. Subhas G, Bhullar JS, Cook J, et al. Topical gentamicin does not provide any additional anastomotic strength when combined with fibrin glue. *Am J Surg*.;201 . Epub ahead of print 2011. DOI: 10.1016/j.amjsurg.2010.09.022.

21. van der Vijver RJ, van Laarhoven CJHM, de Man BM, et al. The effect of fibrin glue on the early healing phase of intestinal anastomoses in the rat. *Int J Colorectal Dis*.;27 . Epub ahead of print 2012. DOI: 10.1007/s00384-012-1435-5.

22. Senol M, Altintas MM, Cevık A, et al. The Effect of Fibrin Glue on the Intensity of Colonic Anastomosis in the Presence and Absence of Peritonitis: An Experimental Randomized Controlled Trial on Rats. *ISRN Surg*.;2013 . Epub ahead of print 2013. DOI: 10.1155/2013/521413.

23. Wu Z, Boersema GSA, Kroese LF, et al. Reducing colorectal anastomotic leakage with tissue adhesive in experimental inflammatory bowel disease. *Inflamm Bowel Dis*.;21 . Epub ahead of print 2015. DOI: 10.1097/MIB.0000000000000336.

24. Stergios K, Frountzas M, Pergialiotis V, et al. The effect of TISSEEL® on colorectal anastomosis healing process in a diabetic animal experimental model. *In Vivo (Brooklyn)*.;34 . Epub ahead of print 2020. DOI: 10.21873/invivo.11820.

25. Yol S, Tekin A, Yilmaz H, et al. Effects of Platelet Rich Plasma on Colonic Anastomosis. *Journal of Surgical Research*.;146 . Epub ahead of print 2008. DOI: 10.1016/j.jss.2007.05.015.

26. Zhou B, Ren J, Ding C, et al. Protection of colonic anastomosis with platelet-rich plasma gel in the open abdomen. *Injury*.;45 . Epub ahead of print 2014. DOI: 10.1016/j.injury.2014.01.018.

27. Daglioglu YK, Duzgun O, Sarici IS, et al. Comparison of platelet rich plasma versus fibrin glue on colonic anastomoses in rats. *Acta Cir Bras*.;33 . Epub ahead of print 2018. DOI: 10.1590/s0102-865020180040000005.

28. Weiss M, Haj M. Gastrointestinal anastomosis with histoacryl glue in rats. *Journal of Investigative Surgery*.;14 . Epub ahead of print 2001. DOI: 10.1080/089419301750072176.

29. Nursal TZ, Anarat R, Bircan S, et al. The effect of tissue adhesive, octyl-cyanoacrylate, on the healing of experimental high-risk and normal colonic anastomoses. *Am J Surg*.;187 . Epub ahead of print 2004. DOI: 10.1016/j.amjsurg.2003.02.007.

30. Irkorucu O, Ucan BH, Cakmak GK, et al. Effect of 2-octyl-cyanoacrylate on ischemic anastomosis of the left colon. *Journal of Investigative Surgery*.;22 . Epub ahead of print 2009. DOI: 10.1080/08941930902866261.

31. Kayaoglu HA, Ersoy OF, Ozkan N, et al. Effect of N-butyl-2-cyanoacrylate on high-risk colonic anastomoses. *Kaohsiung Journal of Medical Sciences*.;25 . Epub ahead of print 2009. DOI: 10.1016/S1607-551X(09)70058-0.

32. Bae KB, Kim SH, Jung SJ, et al. Cyanoacrylate for colonic anastomosis; is it safe? *Int J Colorectal Dis*.;25 . Epub ahead of print 2010. DOI: 10.1007/s00384-009-0872-2.

33. Wu Z, Vakalopoulos KA, Boersema GSA, et al. The prevention of colorectal anastomotic leakage with tissue adhesives in a contaminated environment is associated with the presence of anti-inflammatory macrophages. *Int J Colorectal Dis*.;29 . Epub ahead of print 2014. DOI: 10.1007/s00384-014-2012-x.

34. Güngör G, Demiral G, Şenol M, et al. Cyanoacrylate application on colonic anastomosis: Is it safe or not? *Prz Gastroenterol*.;11 . Epub ahead of print 2016. DOI: 10.5114/pg.2016.57737.

35. Vakalopoulos KA, Wu Z, Kroese LF, et al. Clinical, mechanical, and immunohistopathological effects of tissue adhesives on the colon: An in-vivo study. *J Biomed Mater Res B Appl Biomater*.;105 . Epub ahead of print 2017. DOI: 10.1002/jbm.b.33621.

36. Vakalopoulos KA, Bosmans JWAM, van Barneveld KWY, et al. Impact of tissue adhesives on the prevention of anastomotic leakage of colonic anastomoses: an in vivo study. *Int J Colorectal Dis*.;32 . Epub ahead of print 2017. DOI: 10.1007/s00384-017-2834-4.

37. Costales AB, Patil D, Mulya A, et al. 2-Octylcyanoacrylate for the prevention of anastomotic leak. *Journal of Surgical Research*.;226 . Epub ahead of print 2018. DOI: 10.1016/j.jss.2018.01.026.

38. Despoudi K, Mantzoros I, Ioannidis O, et al. Effects of albumin/glutaraldehyde glue on healing of colonic anastomosis in rats. *World J Gastroenterol*.;23 . Epub ahead of print 2017. DOI: 10.3748/wjg.v23.i31.5680.

39. Ustek S, Kismet K, Akkus MA, et al. Effect of povidone-iodine liposome hydrogel on colonic anastomosis. *European Surgical Research*.;37 . Epub ahead of print 2005. DOI: 10.1159/000087870.

40. Cueto J, Barrientos T, Rodríguez E, et al. A New Biodegradable Adhesive for Protection of Intestinal Anastomoses. Preliminary Communication. *Arch Med Res*.;42 . Epub ahead of print 2011. DOI: 10.1016/j.arcmed.2011.09.007.

41. Deng Y, Ren J, Chen G, et al. Tannin-based adhesive for protection of colonic anastomosis in the open abdomen. *J Biomater Sci Polym Ed*.;28 . Epub ahead of print 2017. DOI: 10.1080/09205063.2016.1268462.

42. Eryilmaz R, Samuk M, Tortum OB, et al. The role of dura mater and free peritoneal graft in the reinforcement of colon anastomosis. *Journal of Investigative Surgery*.;20 . Epub ahead of print 2007. DOI: 10.1080/08941930601126108.

43. Ozel SK, Kazez A, Akpolat N. Does a fibrin-collagen patch support early anastomotic healing in the colon? An experimental study. *Tech Coloproctol*.;10 . Epub ahead of print 2006. DOI: 10.1007/s10151-006-0285-y.

44. Hoeppner J, Willa K, Timme S, et al. Reinforcement of colonic anastomoses with a collagenous double-layer matrix extracted from porcine dermis. *European Surgical Research*.;45 . Epub ahead of print 2010. DOI: 10.1159/000318856.

45. Hoeppner J, Wassmuth B, Marjanovic G, et al. Anastomotic sealing by extracellular matrices (ECM) improves healing of colonic anastomoses in the critical early phase. *Journal of Gastrointestinal Surgery*.;14 . Epub ahead of print 2010. DOI: 10.1007/s11605-010-1191-1.

46. Schreinemacher MH, Bloemen JG, van der Heijden SJ, et al. Collagen fleeces do not improve colonic anastomotic strength but increase bowel obstructions in an experimental rat model. *Int J Colorectal Dis*.;26 . Epub ahead of print 2011. DOI: 10.1007/s00384-011-1158-z.

47. Sabino FD, Campos CFF, Caetano CER, et al. Effects of TachoSil and 5-fluorouracil on colonic anastomotic healing. *Journal of Surgical Research*.;192 . Epub ahead of print 2014. DOI: 10.1016/j.jss.2014.05.067.

48. Suárez-Grau JM, Bernardos García C, Cepeda Franco C, et al. Fibrinogen-thrombin collagen patch reinforcement of high-risk colonic anastomoses in rats. *World J Gastrointest Surg*.;8 . Epub ahead of print 2016. DOI: 10.4240/wjgs.v8.i9.627.

49. Henne-Bruns D, Kreischer HP, Schmiegelow P, et al. Reinforcement of colon anastomoses with polyglycolic acid mesh: An experimental study. *European Surgical Research*.;22 . Epub ahead of print 1990. DOI: 10.1159/000129105.

50. Uludag M, Citgez B, Ozkaya O, et al. Effects of amniotic membrane on the healing of normal and high-risk colonic anastomoses in rats. *Int J Colorectal Dis*.;24 . Epub ahead of print 2009. DOI: 10.1007/s00384-009-0691-5.

51. Sukho P, Boersema GSA, Cohen A, et al. Effects of adipose stem cell sheets on colon anastomotic leakage in an experimental model: Proof of principle. *Biomaterials*.;140 . Epub ahead of print 2017. DOI: 10.1016/j.biomaterials.2017.06.011.

52. Nakamura T, Yokoyama U, Kanaya T, et al. Multilayered Human Skeletal Muscle Myoblast Sheets Promote the Healing Process After Colonic Anastomosis in Rats. *Cell Transplant*.;30 . Epub ahead of print 2021. DOI: 10.1177/09636897211009559.

53. Moazami N, Oz MC, Bass LS, et al. Reinforcement of Colonic Anastomoses With a Laser and Dye–Enhanced Fibrinogen. *Archives of Surgery*.;125 . Epub ahead of print 1990. DOI: 10.1001/archsurg.1990.01410230046008.

54. Giuratrabocchetta S, Rinaldi M, Cuccia F, et al. Protection of intestinal anastomosis with biological glues: An experimental randomized controlled trial. *Tech Coloproctol*.;15 . Epub ahead of print 2011. DOI: 10.1007/s10151-010-0674-0.

55. Vuocolo T, Haddad R, Edwards GA, et al. A Highly Elastic and Adhesive Gelatin Tissue Sealant for Gastrointestinal Surgery and Colon Anastomosis. *Journal of Gastrointestinal Surgery*.;16 . Epub ahead of print 2012. DOI: 10.1007/s11605-011-1771-8.

56. Medina M, Paddock HN, Connolly RJ, et al. Novel antiadhesion barrier does not prevent anastomotic healing in a rabbit model. *Journal of Investigative Surgery*.;8 . Epub ahead of print 1995. DOI: 10.3109/08941939509023140.

57. Aysan E, Dincel O, Bektas H, et al. Polypropylene mesh covered colonic anastomosis. Results of a new anastomosis technique. *International Journal of Surgery*.;6 . Epub ahead of print 2008. DOI: 10.1016/j.ijsu.2008.04.001.

58. Oka H, Harrison RC, Burhenne HJ. Effect of a biologic glue on the leakage rate of experimental rectal anastomoses. *The American Journal of Surgery*.;143 . Epub ahead of print 1982. DOI: 10.1016/0002-9610(82)90162-3.

59. Libutti SK, Oz MC, Forde KA, et al. Canine colonic anastomoses reinforced with dye-enhanced fibrinogen and a diode laser. *Surg Endosc*.;4 . Epub ahead of print 1990. DOI: 10.1007/BF00591269.

60. Cueto J, Barrientos T, Rodriguez E, et al. Further experimental studies on a biodegradable adhesive for protection of colorectal anastomosis. *Arch Med Res*.;45 . Epub ahead of print 2014. DOI: 10.1016/j.arcmed.2014.03.007.

61. Hagerman GF, Gaertner WB, Ruth GR, et al. Bovine pericardium buttress reinforces colorectal anastomoses in a canine model. *Dis Colon Rectum*.;50 . Epub ahead of print 2007. DOI: 10.1007/s10350-007-0212-y.

62. Gaertner WB, Hagerman GF, Potter MJ, et al. Experimental evaluation of a bovine pericardium-derived collagen matrix buttress in ileocolic and colon anastomoses. *J Biomed Mater Res B Appl Biomater*.;92 . Epub ahead of print 2010. DOI: 10.1002/jbm.b.31488.

63. Gulati SM, Thusoo TK, Kakar A, et al. Comparative study of free omental, peritonenal, Dacron velour, and Marlex mesh reinforcement of large-bowel anstomosis - An experimental study. *Dis Colon Rectum*.;25 . Epub ahead of print 1982. DOI: 10.1007/BF02564157.

64. Trowbridge PE, Howes EL. Reinforcement of colon anastomoses using polyurethane foam treated with neomycin. An experimental study. *The American Journal of Surgery*.;113 . Epub ahead of print 1967. DOI: 10.1016/0002-9610(67)90230-9.

65. Wenger FA, Szucsik E, Hoinoiu BF, et al. Circular anastomotic experimental fibrin sealant protection in deep colorectal anastomosis in pigs in a randomized 9-day survival study. *Int J Colorectal Dis*.;30 . Epub ahead of print 2015. DOI: 10.1007/s00384-015-2260-4.

66. Nordentoft T. Sealing of gastrointestinal anastomoses with fibrin glue coated collagen patch. *Dan Med J*.;62.

67. Wenger FA, Szucsik E, Hoinoiu BF, et al. Is Circular Fibrin Sealing of Low Rectal Anastomosis Able to Prevent Leakage in 21-Day Follow-up? Randomized Experimental Trial in Pigs. *Surg Innov*.;26 . Epub ahead of print 2019. DOI: 10.1177/1553350619834786.

68. Dauser B, Heitland W, Bader FG, et al. Histologic changes in early colonic anastomotic healing using autologous platelet-rich fibrin matrix. *European Surgery - Acta Chirurgica Austriaca*.;52 . Epub ahead of print 2020. DOI: 10.1007/s10353-019-0578-9.

69. Tebala GD, Ceriati F, Ceriati E, et al. The use of cyanoacrylate tissue adhesive in high-risk intestinal anastomoses. *Surg Today*.;25 . Epub ahead of print 1995. DOI: 10.1007/BF00311697.

70. Wu Z, Vakalopoulos KA, Kroese LF, et al. Reducing anastomotic leakage by reinforcement of colorectal anastomosis with cyanoacrylate glue. *European Surgical Research*.;50 . Epub ahead of print 2013. DOI: 10.1159/000350383.

71. Boersema GSA, Vennix S, Wu Z, et al. Reinforcement of the colon anastomosis with cyanoacrylate glue: a porcine model. *Journal of Surgical Research*.;217 . Epub ahead of print 2017. DOI: 10.1016/j.jss.2017.05.001.

72. Yael Nir YK. A Gelatin-Based Prophylactic Sealant for Bowel Wall Closure, Initial Evaluation in Mid-rectal Anastomosis in a Large Animal Model. *J Gastrointest Dig Syst*.;05 . Epub ahead of print 2015. DOI: 10.4172/2161-069x.1000258.

73. Hoeppner J, Crnogorac V, Marjanovic G, et al. Small intestinal submucosa for reinforcement of colonic anastomosis. *Int J Colorectal Dis*.;24 . Epub ahead of print 2009. DOI: 10.1007/s00384-009-0637-y.

74. Testini M, Gurrado A, Portincasa P, et al. Bovine pericardium patch wrapping intestinal anastomosis improves healing process and prevents leakage in a pig model. *PLoS One*.;9 . Epub ahead of print 2014. DOI: 10.1371/journal.pone.0086627.

75. García-Vásquez C, Gómez García De Las Heras S, Pastor Idoate C, et al. Histopathological changes associated to an absorbable fibrin patch (Tachosil®) covering in an experimental model of high-risk colonic anastomoses. *Histol Histopathol*.;33 . Epub ahead of print 2018. DOI: 10.14670/HH-11-930.

76. Rosendorf J, Horakova J, Klicova M, et al. Experimental fortification of intestinal anastomoses with nanofibrous materials in a large animal model. *Sci Rep*.;10 . Epub ahead of print 2020. DOI: 10.1038/s41598-020-58113-4.

77. Rosendorf J, Klicova M, Cervenkova L, et al. Reinforcement of colonic anastomosis with improved ultrafine nanofibrous patch: Experiment on pig. *Biomedicines*.;9 . Epub ahead of print 2021. DOI: 10.3390/biomedicines9020102.

78. Rosendorf J, Klicova M, Cervenkova L, et al. Double-layered nanofibrous patch for prevention of anastomotic leakage and peritoneal adhesions, experimental study. *In Vivo (Brooklyn)*.;35 . Epub ahead of print 2021. DOI: 10.21873/INVIVO.12314.

79. Maruya Y, Kanai N, Kobayashi S, et al. Autologous adipose-derived stem cell sheets enhance the strength of intestinal anastomosis. *Regen Ther*.;7 . Epub ahead of print 2017. DOI: 10.1016/j.reth.2017.06.004.
