# Supplementary figures and images for: Clinical results after external reinforcement of colorectal anastomosis: a systematic review
Source: Int J Surg. 2023 Sep 13;109(12):4322–32. doi: 10.1097/JS9.0000000000000747 (PMC10720808; doi:10.1097/JS9.0000000000000747)

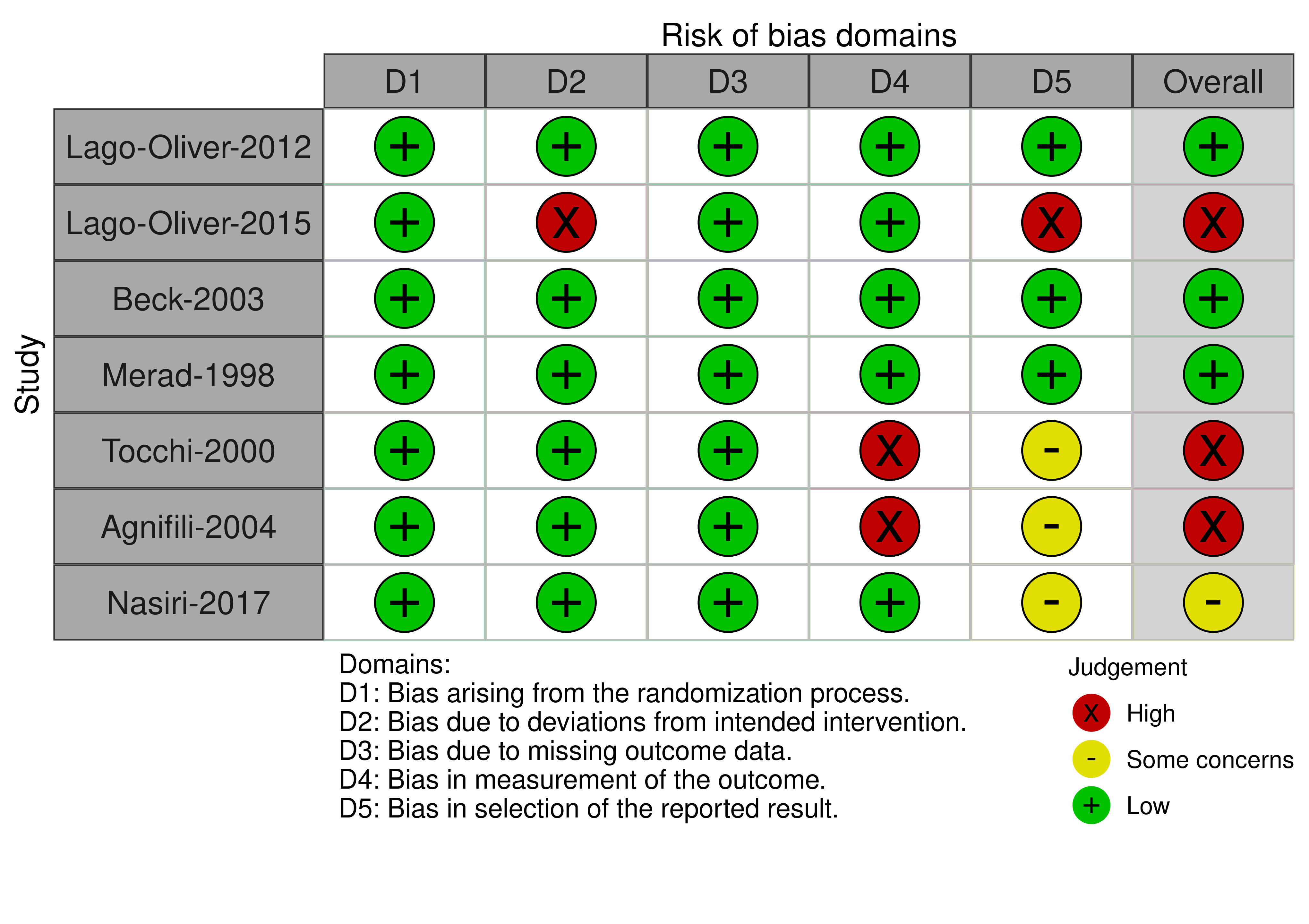

Supplement: SUPPLEMENTARY MATERIAL [file js9-109-4322-s004.jpg]

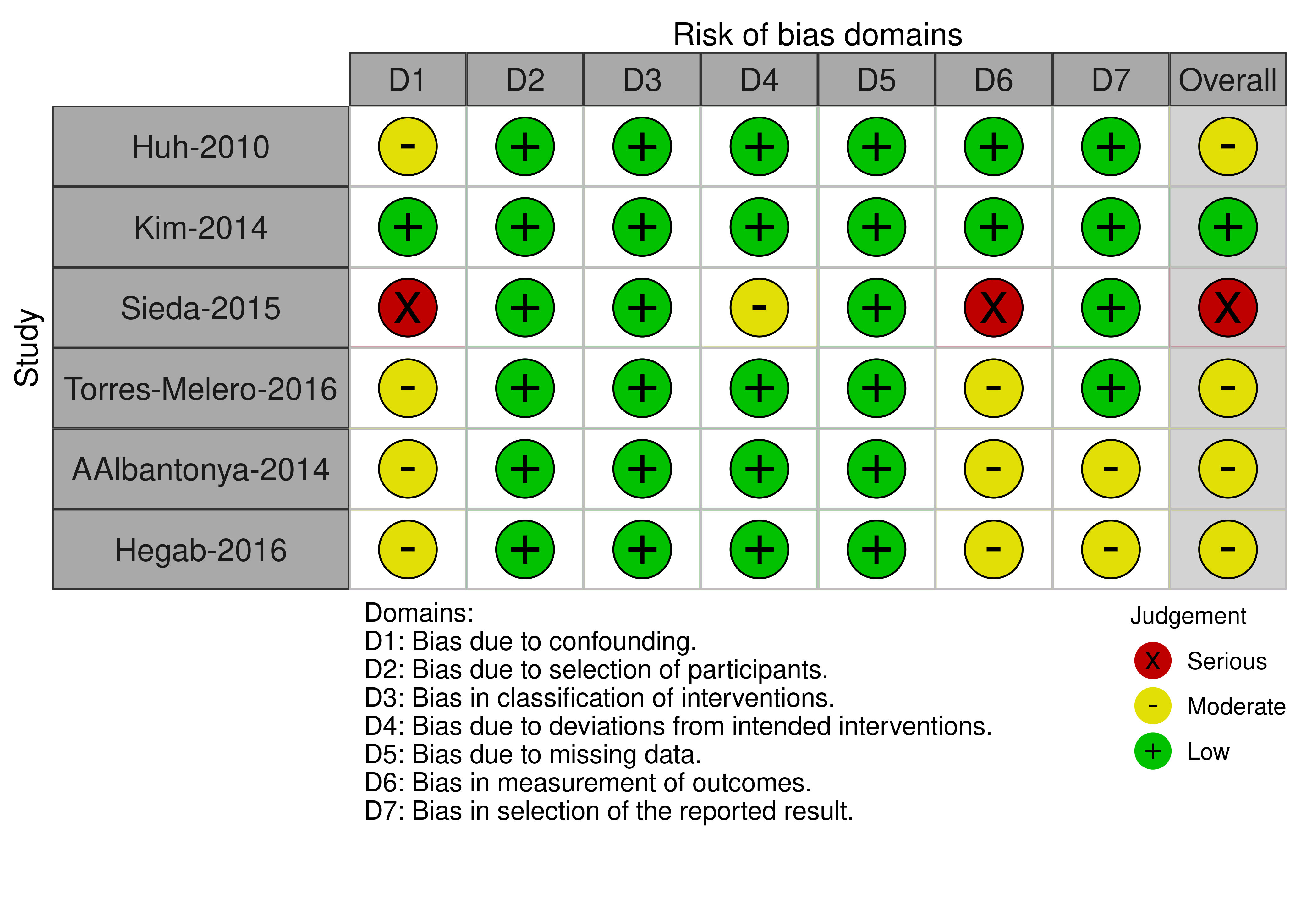

Supplement: SUPPLEMENTARY MATERIAL [file js9-109-4322-s005.jpg]
